# Data and data privacy impact assessments in the context of AI research and practice in the UK

**DOI:** 10.3389/frhs.2025.1525955

**Published:** 2025-04-16

**Authors:** Fiona J. Gilbert, Jo Palmer, Nick Woznitza, Jonathan Nash, Carla Brackstone, Lisa Faria, J. Kevin Dunbar, Henry David Jeffry Hogg, Xiaoxuan Liu, Alastair K. Denniston

**Affiliations:** ^1^ Department of Radiology, Clinical School, University of Cambridge, Cambridge, United Kingdom; ^2^ Department of Inflammation and Ageing AI, University Hospitals Birmingham NHS Foundation Trust, Birmingham, United Kingdom; ^3^ School of Allied and Public Health, UCL, University College London Hospitals NHS Foundation Trust, London, United Kingdom; ^4^ School of Allied and Public Health, Canterbury Christ Church University, Canterbury, United Kingdom; ^5^ Kheiron Medical Technologies, London, United Kingdom; ^6^ Radiology Department, University Hospitals Sussex NHS Foundation Trust, Brighton, United Kingdom; ^7^ British Society of Breast Radiology, London, United Kingdom; ^8^ Screening Quality Assurance Service (SQAS) - South, NHS England, England, United Kingdom; ^9^ Department of Applied Health Research, School of Medical Sciences, College of Medicine and Health, University of Birmingham, Birmingham, United Kingdom

**Keywords:** artificial intelligence, healthcare, data protection impact assessments, implementation, governance

## Abstract

Artificial intelligence (AI) projects in healthcare research and practice require approval from information governance (IG) teams within relevant healthcare providers. Navigating this approval process has been highlighted as a key challenge for AI innovation in healthcare by many stakeholders focused on the development and adoption of AI. Data privacy and impact assessments are a part of the approval process which is often identified as the focal point for these challenges. This perspective reports insights from a multidisciplinary workshop aiming to characterise challenges and explore potential solutions collaboratively. Themes around the variation in AI technologies, governance processes and stakeholder perspectives arose, highlighting the need for training initiatives, communities of practice and the standardization of governance processes and structures across NHS Trusts.

## 1 Introduction

Ethical practices for protecting personal data help to build trust between patients and the healthcare providers that control their data. Healthcare providers often act as data controllers and have a legal duty to comply with data protection legislation. As a result, they must have robust processes in place to ensure compliance both in the context of research and service evaluation or provision. Data Protection Impact Assessments (DPIAs) form one of these processes, required by the 2018 Data Protection Act to be undertaken by data controllers (1). Data controllers have DPIAs in place for routine data processing activities, but also establish processes to check that new projects are in accordance with them (2). Projects involving artificial intelligence (AI) technologies often diverge from the processes described in DPIAs for routine data processing activities, requiring a new project-specific DPIA to be completed. Furthermore, the variable performance of AI technologies across different geographical and temporal settings requires evaluation to be performed and repeated in many healthcare providers.

As a result, requirements for project (and data controller) specific DPIAs are often encountered by advocates of clinical AI as they seek to use patient data for various stages of AI development, evaluation and use. An important distinction of these AI projects that influences the number and variety of DPIAs required is whether the project is research and which organization is acting as sponsor for that research. This is because when data is being used for research purposes, the NHS Healthcare Research Authority recognizes the sponsor of that research as data controller for the project. Even when multiple healthcare providers are contributing data for research as study sites, it is the sponsor organization that takes responsibility for this single DPIA (3). Contrastingly, in projects defined as local audit, service evaluation or clinical use of AI, each healthcare provider undertaking the project will be an independent data controller and require its own DPIA. DPIAs require approval from information governance (IG) professionals within the data controller organization before the proposed project can go ahead. A DPIA is a requirement if processing of personal data is likely to result in high risk to those individuals and is considered good practice for any major project processing large volumes of personal data. Although IG professionals may be used to assessing risk and undertaking DPIA for conventional interventions and research (such as clinical trials of a drug), AI health technologies provide new challenges for assessing risk both due to their unfamiliarity as a class of intervention and the extensive use of data, but also due to the wide variation in individual product function, clinical risk, and use of personal data.

The Incubator for AI and Digital Healthcare is an NIHR-supported cross-sector community which is working collaboratively to support responsible innovation in AI health technologies and their safe implementation into the NHS (4). The Incubator was approached by a number of clinical AI experts who had recent experience delivering multicentre AI trials within the NHS and had encountered significant unexpected challenges relating to data governance including delays in approval of the DPIA within these studies. In response the Incubator brought together UK leaders in AI and in IG to participate in a workshop focusing on DPIAs in the context of AI research and practice to explore important issues. The workshop was chaired by Professor Fiona Gilbert (Lead Proponent), with the Incubator Directors, Professor Alastair Denniston and Dr Xiaoxuan Liu. Participants were invited based on their involvement in designing, researching or implementing AI health technologies or for their role in IG within the NHS, or for their contribution to national policy in health research.

The workshop was designed around first identifying challenges and then collaboratively exploring solutions. Discussions began with specific real world case studies, comprising three multicentre trials of AI health technologies and their pathway towards delivery. This pathway included addressing data governance issues, DPIA completion and securing IG approval prior to study commencement. In this perspective, we share challenges that emerged and opportunities for stakeholders in clinical AI innovation to find solutions together.

## 2 Identifying the challenges

**Figure 1:**
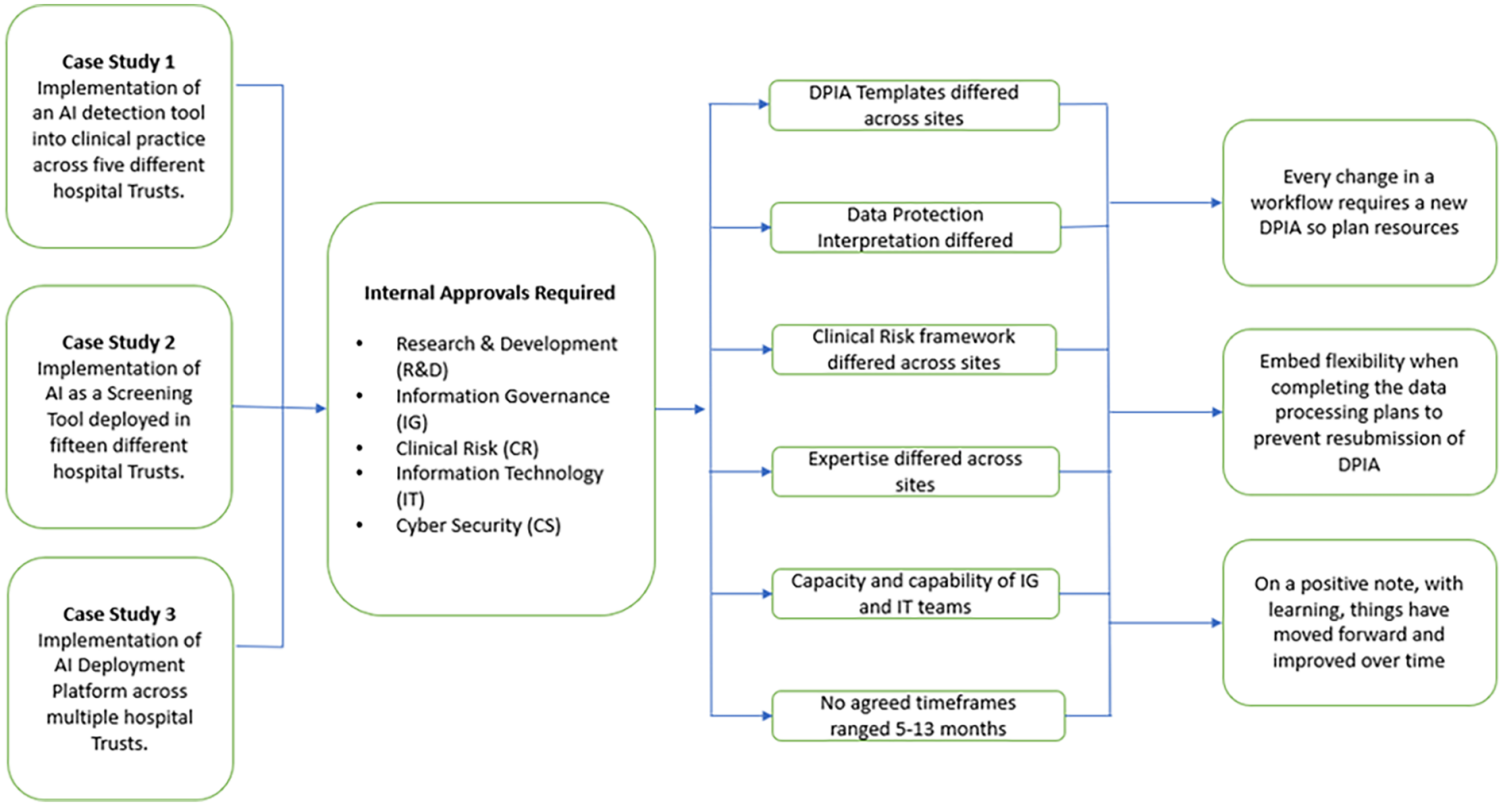
Case studies and lessons learned from gaining approvals from healthcare providers to implement or evaluate clinical AI.

The three case studies were presented by the study teams, comprising clinical, academic and industry representatives. These individuals shared their experience of deploying AI technology across multiple NHS providers (Figure 1). These case studies formed the basis of questions, comments and debate among all participants.

### 2.1 It's not all about the DPIA

Participants, both AI experts and IG experts, noted that there is a need to recognize that the DPIA forms just one part of the process of securing local approval to undertake a research study with an AI health technology. Two critical and linked themes that emerged were that when it comes to running these trials, first achieving local approval is not just about the IG requirements (key point 1); and, second, receiving IG approval is not just about the DPIA (key point 2).

Outside of IG specific considerations and DPIA, the case-studies noted that a robust local approval process would consider clinical safety, technical feasibility, cyber security and potentially other assessments (e.g., wider system impacts, issues of equity etc). Whilst projects involving many intervention types require coordinated assessments of these different risk domains, the breadth and complexity of the assessments required for approval appears as a distinguishing feature of those involving AI. These risk assessment functions may be siloed between different officers or committees within the provider institution and may have complex interdependencies, without a clear hierarchy of approval which may add to uncertainty and delay. The individuals championing clinical AI may have very little awareness of these different processes, and it seems common for them to view these complex assessments of risk as simply an “IG issue”, to be addressed through the DPIA.

A third theme that emerged was about improving understanding and communication (key point 3). For the researcher who seeks to implement an AI technology locally, it is important not to attribute cumulative frustrations with a local governance process to DPIAs or to think of IG professionals as “blockers”, when they are a key component of a provider's “enablement” services. It was noted that such language often shows a lack of understanding of the process, the decisions being made, and is not a productive basis for the collaborative working relationship required. It was highlighted that IG professionals “have more to lose than they have to gain” with these decisions: specifically they take on personal and organisational risk by issuing approvals, but rarely gain any recognition for the responsible innovation they enable. Champions of AI may experience variation in approval processes for an identical technology as inexplicable and time-consuming. This can be better understood as independent data controllers responsibly accommodating the significant variation to risk brought in by differences in the technology's proposed use, host digital infrastructure and institutions' own cultures of leadership and practice. The decentralised responsibility of each data controller to absorb these risks locally is a fundamental limit to the level of coordination and speed that can be expected from the approval process. This may be mitigated by a stronger role for central guidance or assessment but improving understanding among stakeholders of one another's scope of practice and communication between them may independently promote more collaborative and effective working.

### 2.2 There is considerable variation in governance processes between health institutions

Drawing upon the case-studies presented, and their wider experience, workshop participants spoke about the wide variation in governance processes between health institutions (key point 4). There were examples of AI vendors and study teams creating a dedicated role for DPIA completion and management. This reflected the time expended completing forms and trying to understand and then navigate governance processes, which differed between every single health institution they onboarded. Challenges include requests for differing (often overlapping) documentation and inconsistencies in the accepted templates for identically titled documentation, due to local adaptations. Navigating this variability was felt to consume a disproportionate amount of the resources attributed to obtaining AI governance approval, and was particularly problematic within research funding paradigms, which often impose time limits in which projects must be completed. The possibility of having a standardised, nation-wide DPIA template has been previously proposed and was considered in this workshop. Such a template has been developed within NHSE but appeared not to have significant uptake so far (5). IG professionals and other participants advised that documents and processes between healthcare providers are unlikely to be standardised for as long as risk and responsibility lie with the institutions in their role as data controllers (key point 5). Differences in the DPIA template and other assessment requirements will often reflect the relative concerns of the Data Controller, their interpretation of the law, and their attitude to risk.

Participants also reported significant variations in the interpretation of data law including GDPR between healthcare providers (key point 6), creating variation in governance that participants found challenging to navigate, and affected the likelihood of approval. Differences between institutions included (1) variation in the interpretation of the roles of data controller and data processor; and (2) the level at which data might be considered to be “effectively anonymized” (and no longer subject to GDPR) vs. still being considered as pseudonymized data (subject to GDPR).

### 2.3 Not all AI – and not all uses of AI - are the same

There are some stages in AI product development where it is less clear whether data processing activities should be considered as research, or part of standard quality assurance or other process (key point 7). This has consequences for the legal basis for data processing purposes. Examples include, local assurance processes (which may include calibration prior to routine deployment) or auditing of clinical AI (ongoing assessment of quality and safety post-deployment). Participants noted variation between different institutions as to whether these evaluations should be considered as research, routine clinical care or some other category.

Some applications of AI may bring additional considerations: for example if AI is being used autonomously, subject rights related to the use of autonomous AI systems must be accommodated. Looking to the future, if continuously learning AI models are used then the simultaneous use of an individual's data for their personal care, model improvement, supporting others' care, and the commercial interests of the vendor must also be considered.

### 2.4 Recognising our blind-spots

**Figure 2:**
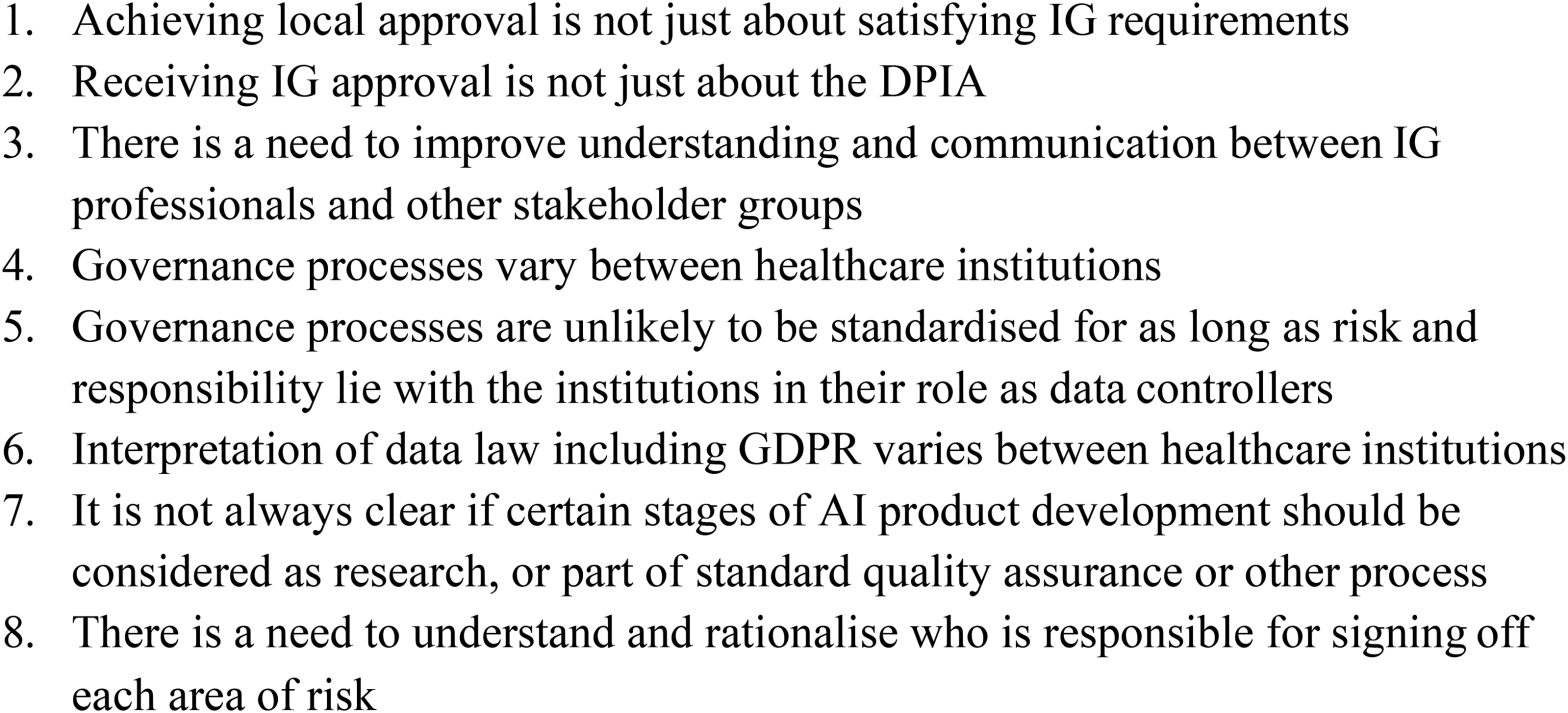
Summary box of key notes from the workshop.

Throughout the workshop there was an increasing awareness of “blind-spots” across the community, and the value of better understanding each other's requirements and more effective communication (key point 8) (Figure 2). For example, the more familiarity and confidence that IG professionals have with different types of AI, the greater their ability to assess the risk of any proposed project (and its technology) correctly and efficiently. It is recognised that with a new technology - in fact AI represents a whole group of new technologies - most IG professionals are currently in a steep phase of a learning curve.

Similarly, the more understanding and respect that AI experts have for the IG process, the greater their ability to articulate and define the data processing activities and responsibilities as part of the DPIA. Resources that were highlighted that may help each group skill up and communicate better include: the ICO AI toolkit (6), BS30440 Validation of AI in Healthcare (7), the AI and Digital Regulations Service (8), the NHSE IG FAQ's (9) and the DPIA templates (5).

## 3 Identifying potential solutions

### 3.1 Who?

The participants considered “what good looks like” for a local approval process for a trial of an AI health technology: specifically, there is a need to understand and rationalise who is responsible for signing off each area of risk, including information governance, clinical safety, technical feasibility, cybersecurity and any other issues. There was consensus about the types of risk being considered, and the professional groups best placed to oversee those risks, but considerable variation was noted between healthcare providers as to the titles of the roles and the structures within which they sat, which made the process of adding additional sites slow and inefficient. A compounding issue is the interdependency of each function, for example, clinical safety assurance may sometimes be considered dependent on having an approved DPIA, and vice versa, leading to a governance grid-lock. This lack of a clear pathway lead one participant to comment that it was easier to run pre-market “first in man” drug studies than undertaking an evaluation of an AI product even when it already had full market approval. To address this issue a pragmatic and shared understanding must be developed across the community about which individuals are responsible for each step of the local governance paradigm. System-wide achievement of this goal would be far less complex if there was better alignment between these local paradigms.

### 3.2 How?

A number of suggestions were made throughout the day and are highlighted here for further consideration.

#### 3.2.1 Principles

• Take a broader view: individuals need to respect and seek to understand the priorities and constraints that others community members are working in**,** so that they can work effectively together to accelerate research that improves patient care.
• Support with education and training: greater understanding and familiarity with clinical AI technologies and governance considerations across the community can support confident, consistent and collaborative decision-making.

#### 3.2.2 Process

• Create an NHS-wide framework for AI governance including assessment of “AI readiness”: an AI Implementation Framework with agreed principles for AI Governance functions (IG, clinical safety, technical feasibility, cybersecurity, and other factors), and consistent distribution of those functions to named roles and structures within healthcare providers would radically improve shared understanding across the ecosystem and improve efficiency for AI research and practice.
• Full standardisation of the IG and AI governance process (and accompanying forms): harmonising forms (notably DPIA form) and having an identical assessment of risk between institutions (possibly assessed nationally) could be advantageous but would require a significant intervention (probably at Department of Health and Social Care level) given that the risk sits with the Data Controller.
• Provide clarity on “borderline” non-research applications: it would be helpful if the Health Research Authority could provide guidance on what governance pathway an AI project (e.g., a service evaluation) falls under when it is not part of a traditional clinical trial nor part of routine clinical care
• Sustain a national community of practice in AI implementation with due emphasis on good governance: it is recognised that effective and efficient governance is a cornerstone of all research, and that responsible introduction of AI health technologies need the AI, IG and clinical communities to work effectively together.

## 4 Discussion

AI health technologies have significant potential to improve healthcare but carry a number of risks that need to be evaluated. As with any technology it is important that the governance process that leads to the decision to use it in the context of research or practice is robust. This requires that decision-makers are provided with the appropriate information and have sufficient understanding of the technology and its intended use to accurately assess any associated risks. It is also reasonable to expect that that decision is consistent across the NHS, and that that decision is made in an efficient and timely manner. The importance of patient and public perspectives in AI governance should not be overlooked and strategies for incorporating their voices would ensure that governance frameworks align with societal needs and expectations. Ethical and equity should also be a consideration to ensure minority groups are not being disadvantaged. This Workshop hosted by the NIHR-supported Incubator in AI & Digital Healthcare enabled stakeholders from the AI, IG and clinical communities to share experiences and characterise factors that influence the efficiency and effectiveness of DPIAs and other governance processes for AI research and practice in the NHS. Given the interdisciplinarity of these factors, it is clear that the community itself will be just as valuable as these insights in enabling progress.

## Data Availability

The original contributions presented in the study are included in the article/Supplementary Material, further inquiries can be directed to the corresponding author.
